# Evaluation the efficacy and safety of simultaneous splenectomy in liver transplantation patients: A meta-analysis

**DOI:** 10.1097/MD.0000000000010087

**Published:** 2018-03-09

**Authors:** Chao He, Xiaojuan Liu, Wei Peng, Chuan Li, Tian-fu Wen

**Affiliations:** a Department of Liver Surgery and Liver Transplantation Center; b Department of Anesthesia, West China Hospital of Sichuan University, Sichuan, China.

**Keywords:** efficacy, liver transplantation, meta-analysis, safety, splenectomy

## Abstract

**Background:**

Simultaneous splenectomy during liver transplantation (LT) is debated. The present meta-analysis assessed the efficacy and safety of splenectomy on the outcome of LT patients.

**Methods:**

We searched PubMed, Embase, and Wanfang databases for relevant studies published until the date of July 15, 2017. Quality assessment of the included studies was performed using a modified Newcastle-Ottawa Scale judgment. The data were analyzed using RevMan5.3 software.

**Results:**

A total of 16 studies consisting of 2198 patients (892 patients received splenectomy during LT [SPLT group] and 1306 patients received LT only [LT group]) were included in the present meta-analysis. Efficacy analysis revealed that pooled hazard ratio for overall survival (OS) between 2 groups was not significantly different (hazard ratio = 1.03; 95% confidence interval [CI]: 0.71–1.50). SPLT group had less postoperative rejection (odds ratio [OR] = 0.63, 95% CI: 0.50–0.79) and small for size syndrome (OR = 0.23, 95% CI: 0.07–0.79). SPLT group had significantly lower preoperative platelet (mean difference [MD] = −17.23, 95% CI: −19.54, −14.91), but significantly higher postoperative platelet (MD = 170.45, 95% CI: 108.33–232.56). Conversely, SPLT group had significant higher preoperative portal pressure (MD = 1.54, 95% CI: 0.75–2.33) and significant lower postoperative portal pressure (MD = −1.17, 95% CI: −2.24, −0.11). Safety analysis revealed that SPLT group had significantly longer operation time (MD = 56.66, 95% CI: 35.96–77.35), more intraoperative blood loss (MD = 1.08, 95% CI: 0.25–1.91), and more intraoperative red blood cell (RBC) transfusion (MD = 3.77, 95% CI: 3.22–4.33). Furthermore, SPLT group had significantly higher incidence of postoperative hemorrhage (OR = 3.07, 95% CI: 1.92–4.91), postoperative thrombosis (OR = 3.63, 95% CI: 1.06–12.45), and perioperative infection (OR = 2.62, 95% CI: 1.76–3.90). In addition, perioperative mortality was significantly higher in the SPLT group (OR = 3.14, 95% CI: 1.31–7.52). Postoperative hospital stay did not differ significantly between 2 groups (OR = −1.75, 95% CI: −3.66–0.16).

**Conclusions:**

Splenectomy benefits LT patients in increasing platelet count. However, splenectomy is a morbid procedure as splenectomy increases operation time, intraoperative blood loss, intraoperative RBC transfusion, and postoperative complications. Splenectomy does not improve OS but increase perioperative mortality. Therefore, splenectomy should be performed only in selective patients.

## 1 Introduction

Liver transplantation (LT) is a curative therapy for most end-stage liver diseases (ESLDs) and selected hepatocellular carcinoma (HCC).^[1]^ Nevertheless, many modifications are necessary during LT. For example, Roux-en-Y choledochojejunostomy is indicated when bile flow is impaired or any evidence of irregularity of bile duct, malignancy, or pre-malignancy is found; reconstruction of veins and arteries by recipient great saphenous vein graft is indicated when the inflow or outflow of the donor liver is impaired.^[2,3]^ Among these modifications, simultaneous splenectomy plays an important role and is performed in various conditions, including portal hypertension (PHT), hypersplenism, splenic artery aneurysm (SAA), large patent splenorenal shunts (SRS), completion of interferon (IFN) therapy against hepatitis C infection, ABO-incompatible (ABO-I) LT, prevention of small for size syndrome (SFSS) and etc.^[4–14]^ However, splenectomy is a morbid procedure, which increases the risk of intraoperative bleeding in the context of splenomegaly, PHT, and coagulation dysfunction.^[13,15]^ Furthermore, there are other complications related to splenectomy, such as higher incidence of portal venous system thrombosis and infection, which adversely affect postoperative survival.^[16–20]^ Therefore, less-invasive alternatives with improved safety have been developed to take the place of splenectomy during adult deceased donor liver transplantation (DDLT), such as splenic artery ligation, splenic artery embolization, or left renal vein ligation to modulate portal flow.^[13,21–24]^ In addition, the advent of oral direct-acting antiviral drugs (DAAs) for HCV-infected patients and new protocols of immune suppression for ABO-I LT has decreased the need for splenectomy.^[6,25,26]^

The importance of spleen has been neglected and spleen has been considered not vital so far, as splenectomy is not often associated with immediate consequences.^[27]^ However, spleen plays important roles in various aspects, for example, production of antibodies, elimination of blood-borne pathogens, particularly encapsulated bacteria, maintenance of peripheral tolerance, storing the circulation platelets and erythrocytes, removing the old platelets and erythrocytes from the blood circulation, and serving as a source of adult multipotent stem cells, such as precursors of β-islet secretory cells. Congenital asplenia and splenectomized individuals are susceptible to infection diseases, the most feared one is overwhelming post-splenectomy infection, which lead to death in a short time period.^[28]^ And furthermore, splenectomy is reported to be associated with increased overall cancer risk, diabetes mellitus, and persistent hypercoagulable state, among others.^[29–31]^

At present, it remains unclear whether simultaneously splenectomy is beneficial for LT patients. The aim of the present meta-analysis is to explore in detail the impact of splenectomy on the outcomes in published series of patients who underwent LT.

## 2 Materials and methods

### 2.1 Search strategy

Literatures published until the date of July 15, 2017 were searched in the PubMed, Embase, and Wan-Fang databases by 2 independent investigators (CH and XJL) using the keywords “splenectomy” AND “liver transplantation.” Pubmed search was conducted by endnote X7 software. Embase and Wan-Fang database were searched on Webpage; finally citations were managed by endnote X7 software with the assist of Excel 2013. The references of each literature were examined to identify appropriate articles. After this initial screening, the database of selected studies was cross-checked to identify discrepancies. If multiple publications from the same cohort were found, data from the most comprehensive report were included. Duplicated literatures were finally removed. Thereafter, review of full-text articles and quality assessment were carried out by the same independent reviewers, and a third reviewer was available to adjudicate on any conflicts arising between the two reviewers. Studies were included in the present meta-analysis according to the following criteria: patients who underwent LT; splenectomy as an exposure interest; available data concerning the outcome of interest. Excluded criteria were: only 1 treatment method was used and no contrastive study was performed; 2 surgical procedures were compared in an animal model; data could not be used for statistical analysis; LT and splenectomy were conducted step by step; basic preoperative situation of the 2 groups was obviously different; no access to full text for quality assessment and data extraction; case reports. Because the data included in our study were extracted from published literatures, no approval was required from the institutional review board and patient consent was not necessary.

### 2.2 Data extraction and quality assessment

Two investigators independently extracted the following data from each study: study characteristics, that is, name of first author, publication year, study region, inclusion period of study, study design, and transplantation type; surgery outcomes, that is, operation time, intraoperative blood loss, intraoperative (red blood cell) RBC transfusion, preoperative platelet, postoperative platelet (1 month after surgery), preoperative portal pressure, postoperative portal pressure (1 month after surgery), hospital stay, postoperative complications (intraperitoneal bleeding, thrombosis, infection rate, rejection rate, SFSS, pancreatic leakage), perioperative mortality, and hazard ratio (HR) for overall survival (OS) after surgery; potential sources of heterogeneity. Disagreements were resolved by discussion or consulting experts. Postoperative complications and mortality were defined as adverse events during the first hospital stay, occurred <1 month after surgery. If necessary, the primary authors were contacted to obtain missing data. A modification of the Newcastle–Ottawa Scale was used as an assessment tool for selection, comparability, and outcome assessment.^[32]^

### 2.3 Statistical analysis

We used Review Manager (RevMan; Version 5.3; Cochrane Collaboration) to pool data. For the time-to-event variables, directly extracting HR with 95% confidence interval (CI) from each study was preferential. When the association between splenectomy and HRs of survival was not reported, HRs were calculated according to the methods described by Parmar et al^[33]^ and Tierney et al.^[34]^ If continuous data were presented as median and range, the mean ± standard deviation was calculated according the methods described by Hozo et al.^[35]^ Continuous variables were compared by weighted mean difference (MD). Category variables were compared by odds ratio (OR). The Mantel-Haenszel Q-statistic was used to assess heterogeneity among the studies, and the *I*^2^ statistic was computed to examine the proportion of total variation in the study estimate due to heterogeneity. *P* > .10 or *P* ≤ .10/*I*^2^ ≤50% was considered to indicate no significant heterogeneity between the trials and a fixed-effect model was selected for analysis in such cases. Conversely, *P* ≤ .10/*I*^2^ >50% was considered to indicate significant heterogeneity, and a random-effect model was used. For the integrated results, *P* < .05 indicated statistical significance. The Begg funnel plots were used to estimate potential publication bias. The significance of the pooled HR, OR, MD was determined using the Z test and a *P* value <.05 was considered as statistically significant.

## 3 Results

### 3.1 Literature search

**Figure 1:**
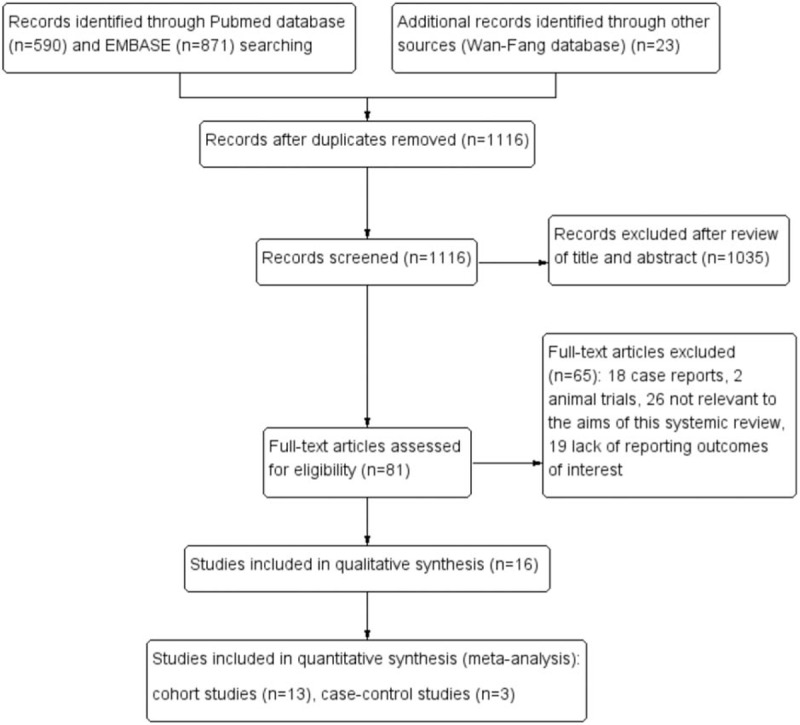
Flowchart showing study identification and selection process.

The search performed in July 2017 identified 1484 citations. Figure 1 shows the process of studies selection according to PRISMA guidelines. After reviewing the titles and authors, 368 duplicates were excluded. A total of 1035 unrelated articles were excluded from the remaining 1116 records after screening the titles and abstracts. After reviewing the full text of the rest of 81 potentially relevant articles, 65 articles were excluded, which include 18 case reports, 2 animal trials, 26 studies not relevant to the aims of this systemic review, and 19 studies lack of outcomes of interest. Sixteen studies were eventually included in the analysis.^[4,5,9,13–15,18–20,36–42]^

### 3.2 Characteristics and quality of included studies

Sixteen studies consisting of 2198 patients (926 patients who had simultaneously splenectomy during LT [SPLT group] and 1393 patients who had LT only [LT group]) finally met eligibility criteria. All of them were retrospective. There were 3 case-control studies and 13 cohort studies. Thirteen studies were performed in the eastern countries (6 in China and 7 in Japan), the other 3 studies were performed in France, Germany, and the United States of America, respectively. Of the 16sixteen included articles, 6 were published before 2010, and the other 10 were published after 2010. The detailed characteristics of the 16 studies, including the first author's names, countries where the studies performed, patient number of 2 groups, periods of inclusion, patient number with HCC, study designs, LT types, and publication years were described in Table 1. The quality of the literatures was assessed using a modification of the Newcastle-Ottawa Scale. Studies given >4 stars were recognized as being moderate to high quality. The results of this assessment are shown in Table 2.

**Table 1:**
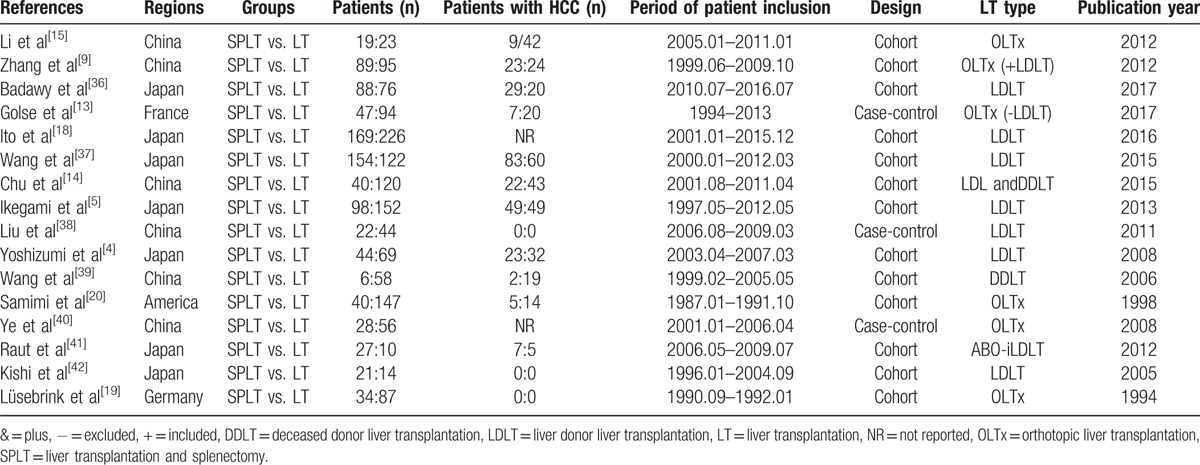
Basic characteristics of all pooled studies in the meta-analysis.

**Table 2:**
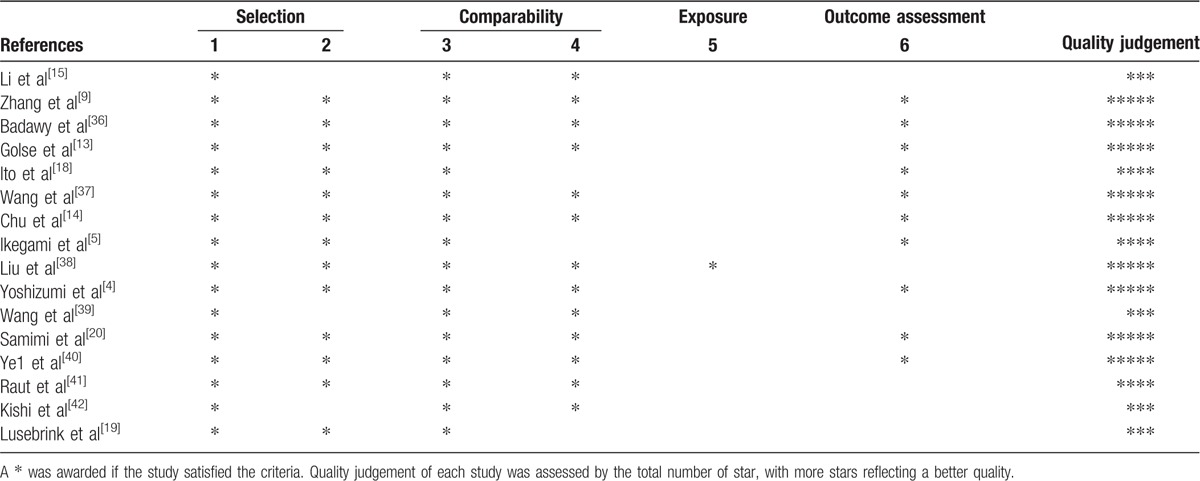
Quality assessment of studies in the meta-analysis based on modified Newcastle–Ottawa Scale judgment.

### 3.3 Efficacy analyses

With respect to efficacy analyses, pooled HR for OS, postoperative rejection, incidence of SFSS, preoperative and postoperative platelet, preoperative portal pressure, and postoperative portal pressure were analyzed. The result of efficacy analysis was demonstrated in Figure 2 . Twelve studies provided information regarding OS, and no study directly provides association of splenectomy and HR of OS. Seven studies provide clear survival curves, which were applicable for HR calculating. The data indicate that the OS between 2 groups was not significantly different (HR = 1.31; 95% CI: 0.81–2.13, *P* = .27, Fig. 2 A). Eleven studies reported postoperative rejection; splenectomy was associated with significantly less rejection (OR = 0.63, 95% CI: 0.50–0.79, *P* < .001, Fig. 2 B). Three studies reported SFSS; the incidence of SFSS was significantly lower in SPLT group (OR = 0.23, 95% CI: 0.07–0.79, *P* = .02, Fig. 2 C). Eight studies reported preoperative platelet; the preoperative platelet was significantly lower in the SPLT group (MD = −17.23, 95% CI: −19.54,−14.91, *P* < .001, Fig. 2 D). Six studies reported postoperative platelet; the postoperative platelet was significantly higher in the SPLT group (MD = 170.45, 95% CI: 108.33–232.56, *P* < .001, Fig. 2 E). Four studies reported preoperative portal pressure and postoperative portal pressurel SPLT group had significant higher preoperative portal pressure (MD = 1.54, 95% CI: 0.75–2.33, *P* < .001, Fig. 2 F) and lower postoperative portal pressure (MD = −1.17, 95% CI: −2.24, −0.11, *P* = .03, Fig. 2 G).

**Figure 2:**
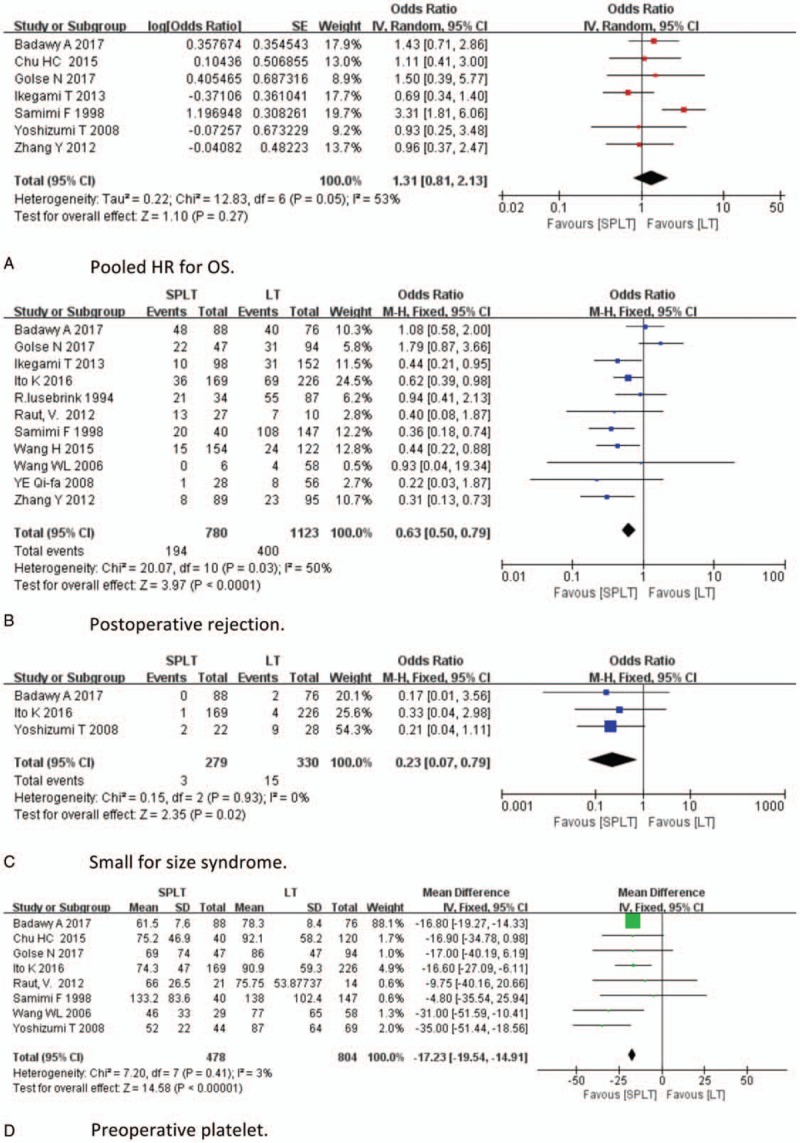
Efficacy analysis.

**Figure 2 (Continued):**
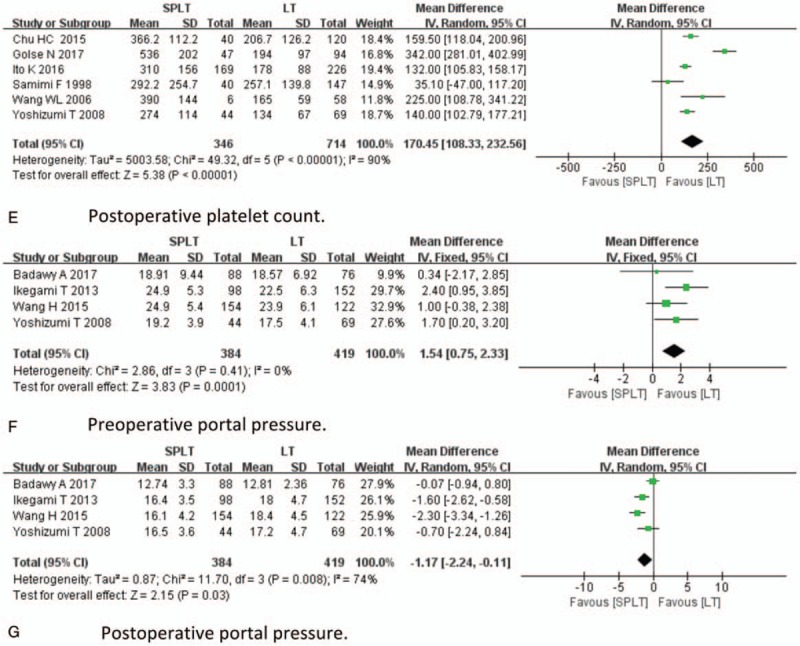
Efficacy analysis.

### 3.4 Safety analysis

With respect to safety analysis, we compared operation time, intraoperative blood loss, intraoperative RBC transfusion, incidence of postoperative hemorrhage, postoperative thrombosis, perioperative infection, perioperative mortality, and postoperative hospital stay of 2 groups. Results of safety analysis were demonstrated in Figure 3 . Twelve studies reported operation time; longer operation time was found be in SPLT group (MD = 56.66, 95% CI: 35.96–77.35, *P* < .001, Fig. 3 A). Eight studies reported intraoperative blood loss; there was no significant difference between 2 groups (MD = 0.78, 95% CI: −0.18–1.74, *P* < .001, Fig. 3 B). Six studies reported intraoperative RBC transfusion. Splenectomy significantly increases intraoperative RBC transfusion (MD = 3.77, 95% CI: 3.22–4.33, *P* < .001, Fig. 3 C). Nine studies reported postoperative hemorrhage; SPLT group had higher incidence of postoperative intraperitoneal bleeding (OR = 3.07, 95% CI: 1.92–4.91, *P* < .001, Fig. 3 D). Eight studies reported postoperative thrombosis, the incidence of which was significantly higher in SPLT group (OR = 3.63, 95% CI: 1.06–12.45, *P* = .04, Fig. 3 E). Seven studies reported perioperative infection, which tended to be higher in SPLT group, but the difference was not significant (OR = 1.96, 95% CI: 0.97–3.97, *P* = .06, Fig. 3 F). Eight study reported perioperative mortality, which was significantly higher in the splenectomy group (OR = 3.14, 95% CI: 1.31–7.52, *P* = .01, Fig. 3 G). Five studies reported postoperative hospital stay, which did not differ significantly between 2 groups (OR = −1.75, 95% CI: −3.66–0.16, *P* = .07, Fig. 3 F). Five studies reported pancreatic leakage after splenectomy, with an incidence of 6.2% (25/406).^[4,5,36–38]^ One study reported pancreatitis, with an incidence of 8.5%.^[13]^

**Figure 3:**
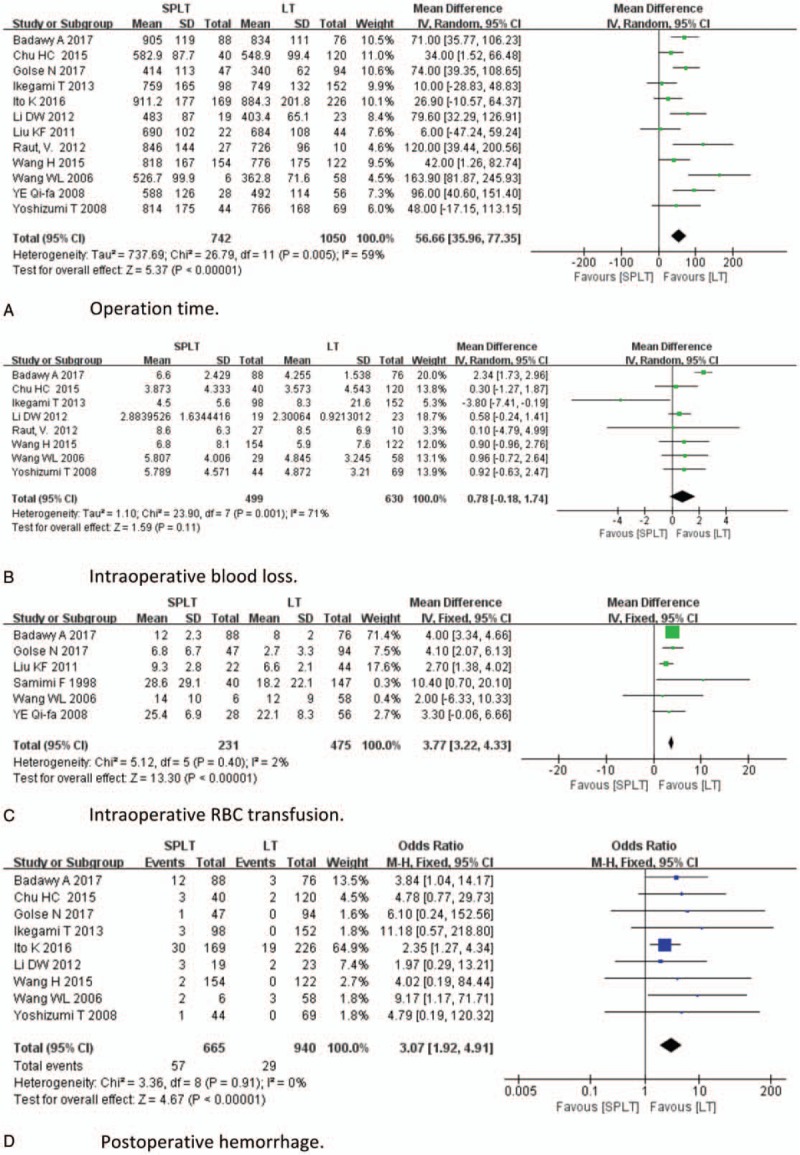
Safety analysis.

**Figure 3 (Continued):**
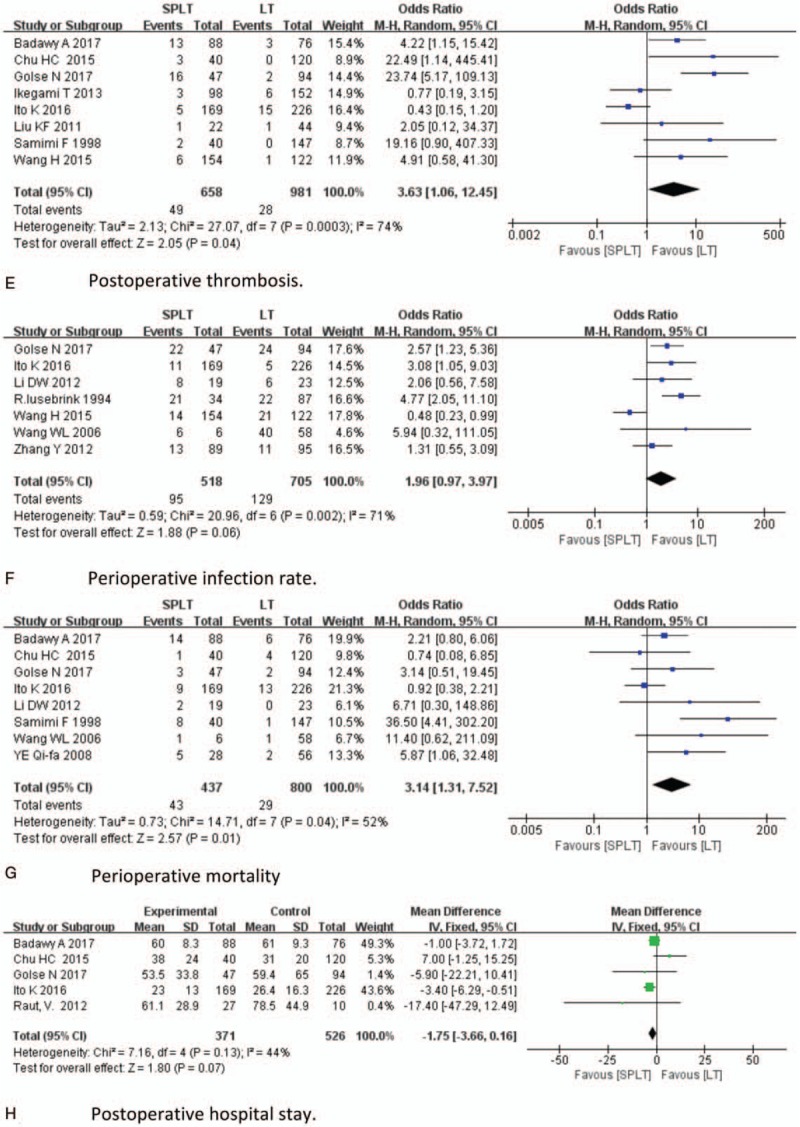
Safety analysis.

### 3.5 Heterogeneity

High heterogeneity was detected for operation time (*I*^2^ = 59%, *P* = .005), intraoperative blood loss (*I*^2^ = 71%, *P* = .001), postoperative thrombosis (*I*^2^ = 74%, *P* = .0004), perioperative infection (*I*^2^ = 71%, *P* = .005), perioperative mortality (*I*^2^ = 52%, *P* = .04), postoperative platelet, (*I*^2^ = 90%, *P* < .001), postoperative portal pressure (*I*^2^ = 74%, *P* = .008), HR for OS (*I*^2^ = 53, *P* = .005).

### 3.6 Sensitivity analyses and publication bias

**Figure 4:**
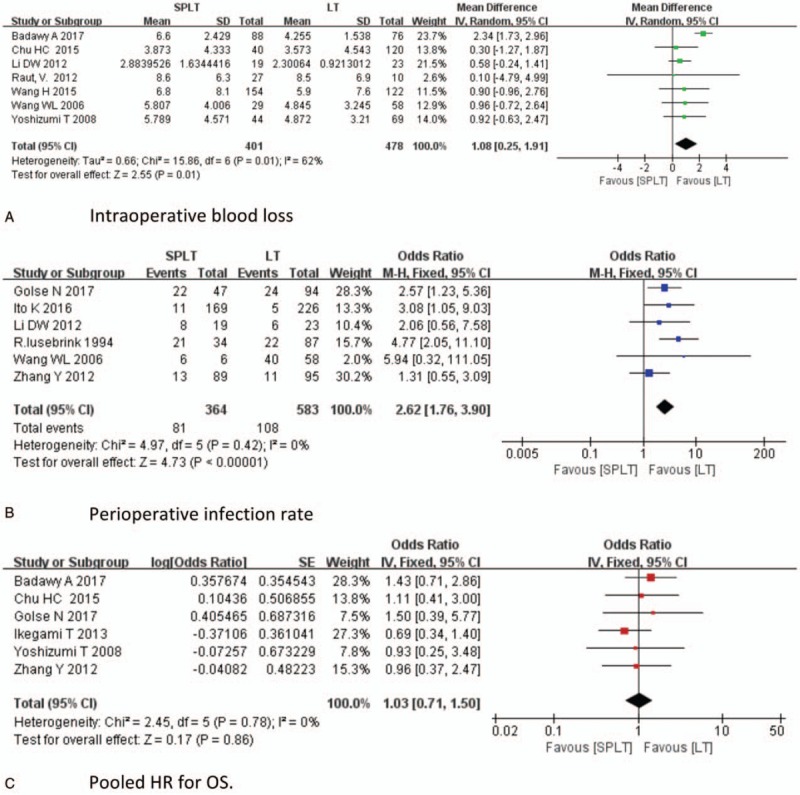
Sensitivity analysis.

Given the detection of heterogeneity between these studies, we conducted sensitivity analyses by omitting each single article in turn. Potential sources of heterogeneity were explored when significant changes of results were found. The results of operation time, intraoperative RBC transfusion, and postoperative hemorrhage, postoperative rejection, perioperative mortality, preoperative platelet, preoperative portal pressure, postoperative platelet, and postoperative portal pressure remained unchanged during the process of removing one study at a time. However, the results of intraoperative blood loss, perioperative infection, and pooled HR for OS were not consistent when performing the sensitivity analysis. The results were demonstrated in Figure 4. Seven studies reported more intraoperative blood loss in SPLT group, whereas 1 study reported less intraoperative blood loss in SPLT group. The baseline characteristics of patients in this study differed significantly.^[5]^ After excluding this study, reanalysis of the remaining 7 studies revealed that splenectomy significantly increases intraoperative blood loss (MD = 1.08, 95% CI: 0.25–1.91, *P* = .01, Fig. 4A). Lower heterogeneity was found in these studies (*I*^2^ = 62%, *P* = .01). Six studies reported that SPLT group had a higher perioperative infection rate than LT group, whereas 1 study reported a lower perioperative infection rate. After excluding this study, no significant heterogeneity was found among remaining studies (*I*^2^ = 0%) and results from the remaining studies suggest that the infection rate remained significantly higher in SPLT group (OR = 2.62, 95% CI: 1.76–3.90, *P* < .001, Fig. 4B). Only 1 study provided a significant HR of OS favoring the LT group, which was published before 2000, whereas the rest of 6 studies, which were published after 2000, demonstrated that the HRs for OS were not significant and with wide ranges. After excluding the study by Samimi, no significant heterogeneity was found among the remaining studies (*I*^2^ = 0%). And the OS of 2 groups did not differ significantly (pooled HR: 1.03, 95% CI: 0.71–1.50, *P* = .86, Fig. 4C). Finally, we created funnel plots for each comparison (Fig. 5 ). These 15 plots were basically inverted and funnel-shaped, with bilateral symmetry, indicating lack of publication bias, and having reliable conclusions.

**Figure 5:**
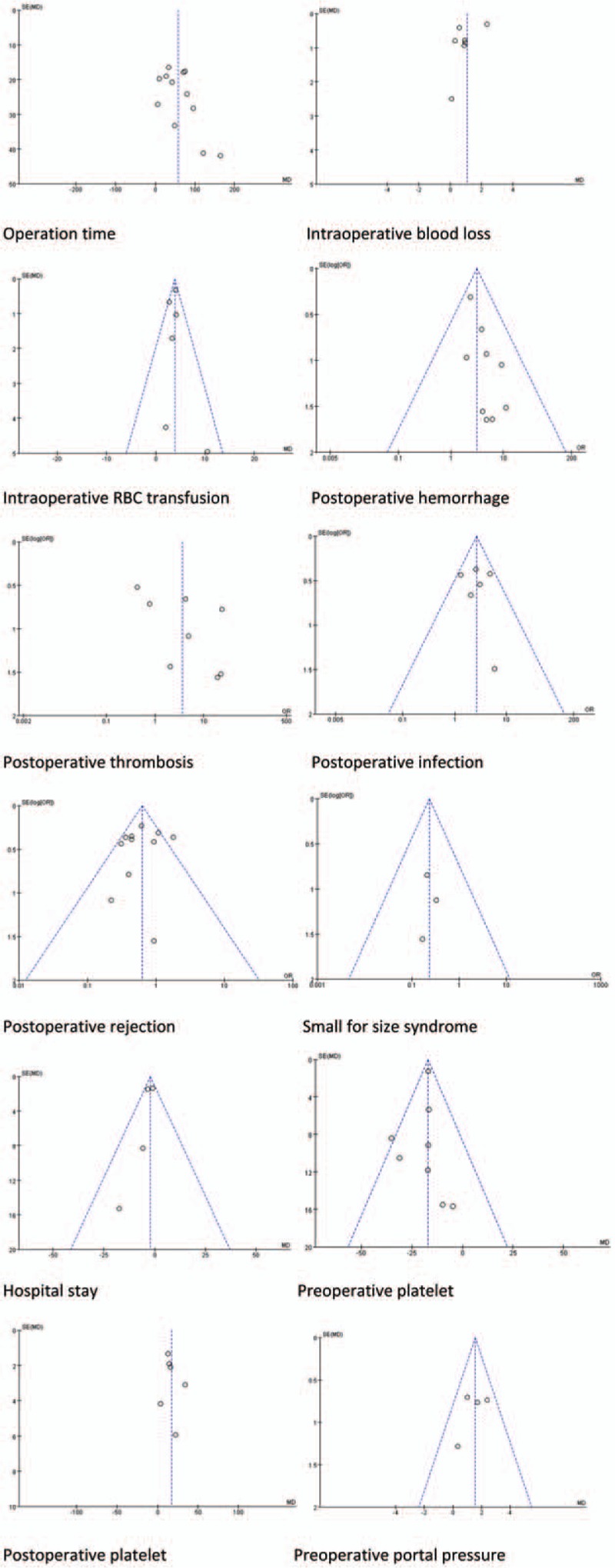
Funnel plots for each comparison.

**Figure 5 (Continued):**
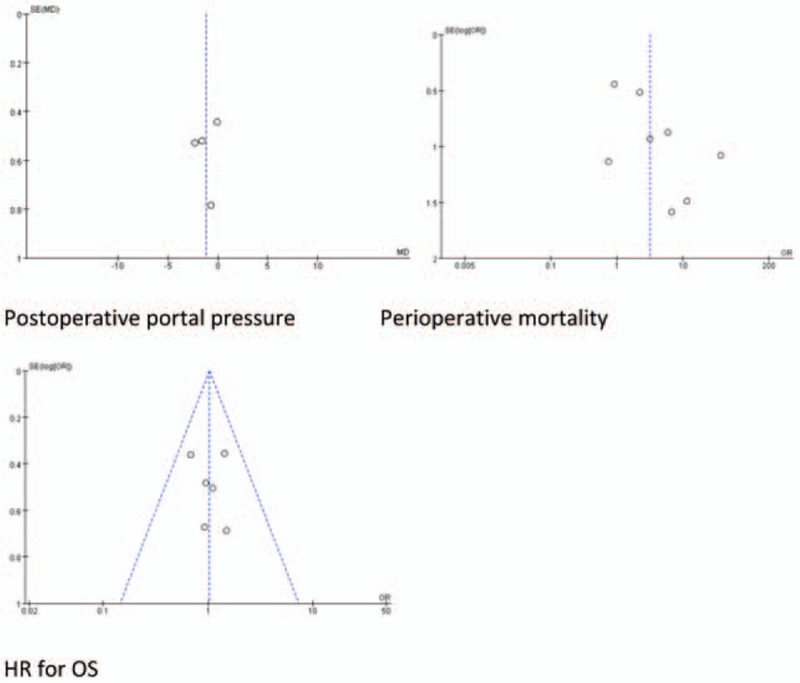
Funnel plots for each comparison.

## 4 Discussion

LT is currently considered a curative therapy for most ESLDs and supported by a grant from selected HCC. However, modifications, such as splenectomy, Roux-en-y anastomosis, and autologous saphenous vein transplantation, are necessary in certain circumstances. Splenectomy which is the most important modification during LT is indicated in various conditions.

First of all, splenectomy is indicated in severe thrombocytopenia. ESLDs and HCCs are often accompanied by splenomegaly, which is secondary to liver cirrhosis and PHT. Hypersplenism is subsequent to splenomegaly and may lead to adverse effects such as pancytopenia.^[43]^ Although LT with a whole liver graft immediately decreases portal pressure, PHT and splenomegaly still persist in some patients; the incidence is even higher in living donor liver transplantation (LDLT) recipients. Low preoperative platelet and the large volume of spleen were reported to be associated with persisting thrombocytopenia.^[44]^ In such circumstances, splenectomy remarkably resolves thrombocytopenia.^[13,18]^ Possible mechanisms may be associated with reduction of the sequestration and destruction of platelets and WBCs in the spleen, and increased release of thrombopoietin.^[45,46]^

Second, simultaneous splenectomy is indicated when using a small-for-size (SFS) graft in the LDLT and split LT settings. The development of LDLT and split LT has dramatically decreased the number of patients on the waiting list and helped to overcome the paucity of liver grafts. But there is a dilemma. Although using smaller grafts is preferred to increase donor safety, SFS graft is the major cause of SFSS, which ultimately leads to liver failure and frequently leads to death.^[47]^ Therefore, a smaller graft with acceptable survival is highly anticipated. Portal hypertension and excess graft inflow were well recognized as the most important predictor of graft dysfunction.^[48]^ To control PHT, several approaches of portal vein pressure (PVP) modulation, other than increasing the graft size, have been proposed. One of these techniques is splenectomy.^[4,49,50]^

The third indication is ABO-I LT. ABO-I LT is generally contraindicated and only performed in a few urgent cases of ESLDs and HCC.^[25,41]^ Poorer outcomes after ABO-I LT compared with ABO compatible LT, such as higher incidence of antibody-mediated rejection (AMR),hepatic arterial thrombosis, biliary complication and cytomegalovirus infection and lower graft survival have been insurmountable barriers to expand its application.^[51,52]^ As vascular, sinusoidal, and biliary epitheliums also express blood group antigens, AMR immediately occurs after ABO-I LT because of the preformed anti-ABO antibodies.^[53]^ Spleen is the site of B-cell maturation and antibodies production; some liver transplant centers developed innovative protocols that include splenectomy as an integral part for ABO-I LT and reported comparable result to ABO-compatible LT.^[54,55]^

The forth indication is to improve the tolerance to IFN therapy for hepatitis C virus (HCV)-infected recipients. Recurrent hepatitis C is the main problem for HCV-infected patients after LT, with a recurrence rate of 11% to 14%, which leads to graft failure in the long term.^[56,57]^ The sustained virologic response ratio with standard IFN and ribavirin therapy for recurrent HCV after transplantation is only approximately 30%.^[42]^ Retransplantation provides a 3-year survival rate of only 40% to 56%.^[58,59]^ One obstacle for starting or continuing combined IFN and ribavirin therapy is blood cytopenia. To overcome this problem, simultaneously splenectomy was performed during LT.^[14]^ Previous studies have reported that SPLT group had significantly higher platelet and leukocyte counts and achieved higher IFN-based anti-viral therapy completion ratio than non-splenectomy group.^[14,42,60]^

Other indications include SAA, large spontaneous SRS, pancreatic tumor, and so on.^[24,61–63]^ Previous study has also reported splenectomy could reduce the incidence of HBV recurrence after LT for HBV-related ESLDs with severe hypersplenism and splenomegaly.^[9]^ Furthermore, splenectomy might be beneficial for preventing autoimmune hepatitis relapse after LT.^[64]^

Although splenectomy may be applied in various conditions, there are oppose opinions toward its use during LT.^[5,13,18–20,36,39]^ Therefore, this meta-analysis aimed at evaluating the efficacy and safety of SPLT for LT patients.

On the one hand, the results of our meta-analysis indicated that when compared to LT group, SPLT group had longer operation time, more intraoperative blood loss and required more intraoperative RBC transfusion. It is explainable as splenectomy increase surgical procedures, and cause more trauma to patients. In addition, the presence of coagulopathy, PHT in ESLDs further increase additional blood lose. Present meta-analysis also revealed that simultaneous splenectomy in LT augments postoperative complications, such as postoperative bleeding, thrombosis, infection and pancreas leakage. And splenectomy also increase perioperative mortality. Therefore, splenectomy is a morbid procedure during LT.

On the other hand, our meta-analysis demonstrated that splenectomy could reduce the incidence of SFSS and postoperative rejection for LT patients, which was a potential protective effect in LT. However, previous studies have reported less invasive methods, such as splenic artery ligation and splenic artery coiling, achieved comparable effects to splenectomy in portal flow modulation and reducing the incidence of SFSS.^[21,49,65,66]^ Furthermore, with improvements in postoperative anti-rejection management such as administration of rituximab and application of plasma exchange, splenectomy has been unnecessary in ABO-I LT setting.^[25]^ Previous studies have reported splenectomy can help preventing HBV recurrence and facilitating IFN therapy in HCV-related LT.^[9,14,60]^ However, management of HBV and HCV has made substantial progress. Fung et al^[67]^ reported that long-term Entecavir monotherapy achieved durable HBsAg seroclearance rate of 92% and undetectable HBV DNA rate of 100% in 8 years after LT. New regimens of DAAs for HCV have been reported to have achieved a sustained virological response rate >90% in LT patients without the need of splenectomy to increase platelet and white blood cell count.^[68]^ Improvement of OS in LT patients is desired; however, the present meta-analysis demonstrated that splenectomy failed to achieve.

There are several limitations of this meta-analysis. First, all the included studies were retrospective researches. Second, the patient number was relative small in each included study. Third, a degree of selection bias has been generated as we excluded studies published in other languages or databases. Fourth, the results of our analysis have some potential confounding factors. For example, indications of LT included benign diseases and malignant diseases, LT types included LDLT and DDLT, and indications of splenectomy differed significantly from each other in the included studies. The power of our results is also compromised in that most studies were not randomized in design, and the basic characteristics of the non-splenectomized group and splenectomized group in some studies were not strictly comparable. However, with only a small number of studies focusing on the outcomes of splenectomy during LT, it is currently impossible to thoroughly perform a meta-analysis evaluating the efficacy and safety of splenectomy in LT patients of a certain disease and with a certain LT type, which is the major origin of bias. Therefore, further well-designed studies are needed to provide more convincing evidence.

In conclusion, simultaneous splenectomy during LT is efficient in increasing platelet count and decreasing portal pressure. However, present meta-analysis reveals that splenectomy tends to increase operation time, intraoperative blood loss, intraoperative RBC transfusion, incidence of postoperative hemorrhage, postoperative thrombosis, and perioperative infection. Splenectomy does not improve OS but increases perioperative mortality. Although splenectomy could reduce the incidence of SFSS and postoperative rejection, there are alternative less invasive therapies. High-efficiency anti-HBV treatment and anti-HCV treatment have rendered splenectomy unnecessary in control HBV and HCV infection. Therefore, splenectomy should be carefully selected to perform in LT patients.

## References

[1] Schilsky ML Moini M Advances in liver transplantation allocation systems. World J Gastroenterol 2016;22:2922–30. 26973389 10.3748/wjg.v22.i10.2922 PMC4779916

[2] Shamsaeefar A Praiss A Podolsky D Biliary reconstruction in liver transplant patients with primary sclerosing cholangitis, duct-to-duct or Roux-en-Y? Clin Transplant 2017;31: 10.1111/ctr.12964 28301681

[3] Wu H Wu H Wu H Using vein grafts in living donor liver transplantation. Zhonghua Gan Zang Bing Za Zhi 2006;14:927–9. 17196139

[4] Yoshizumi T Taketomi A Soejima Y The beneficial role of simultaneous splenectomy in living donor liver transplantation in patients with small-for-size graft. Transpl Int 2008;21:833–42. 18482177 10.1111/j.1432-2277.2008.00678.x

[5] Ikegami T Yoshizumi T Soejima Y Application of splenectomy to decompress portal pressure in left lobe living donor liver transplantation. Fukuoka Igaku Zasshi 2013;104:282–9. 24364263

[6] Bhamidimarri KR Satapathy SK Martin P Hepatitis C virus and liver transplantation. Gastroenterol Hepatol (N Y) 2017;13:214–20. 28546792 PMC5441022

[7] Ikegami T Soejima Y Taketomi A Hypersplenism after living donor liver transplantation. Hepatogastroenterology 2009;56:778–82. 19621701

[8] Cescon M Sugawara Y Takayama T Role of splenectomy in living-donor liver transplantation for adults. Hepatogastroenterology 2002;49:721–3. 12063978

[9] Zhang Y Yan L Wen T Prophylaxis against hepatitis B virus recurrence after liver transplantation for hepatitis B virus-related end-stage liver diseases with severe hypersplenism and splenomegaly: role of splenectomy. J Surg Res 2012;178:478–86. 22483806 10.1016/j.jss.2012.02.047

[10] Heestand G Sher L Lightfoote J Characteristics and management of splenic artery aneurysm in liver transplant candidates and recipients. Am Surg 2003;69:933–40. 14627251

[11] Esquivel CO Klintmalm G Iwatsuki S Liver transplantation in patients with patent splenorenal shunts. Surgery 1987;101:430–2. 3551166 PMC2974321

[12] Sadamori H Yagi T Matsukawa H The outcome of living donor liver transplantation with prior spontaneous large portasystemic shunts. Transpl Int 2008;21:156–62. 18005086 10.1111/j.1432-2277.2007.00593.x

[13] Golse N Mohkam K Rode A Splenectomy during whole liver transplantation: a morbid procedure which does not adversely impact long-term survival. HPB (Oxford) 2017;19:498–507. 28233673 10.1016/j.hpb.2017.01.020

[14] Chu HC Hsieh CB Hsu KF Simultaneous splenectomy during liver transplantation augments anti-viral therapy in patients infected with hepatitis C virus. Am J Surg 2015;209:180–6. 24928331 10.1016/j.amjsurg.2014.03.004

[15] Li DW Du CY Fan B Impact of simultaneous splenectomy and orthotopic liver transplantation in patients with end-stage liver diseases and splenic hyperfunction. Hepatobiliary Pancreat Dis Int 2012;11:489–93. 23060393 10.1016/s1499-3872(12)60212-4

[16] Neumann UP Langrehr JM Kaisers U Simultaneous splenectomy increases risk for opportunistic pneumonia in patients after liver transplantation. Transpl Int 2002;15:226–32. 12012043 10.1007/s00147-002-0399-8

[17] Qi X Han G Ye C Splenectomy causes 10-fold increased risk of portal venous system thrombosis in liver cirrhosis patients. Medical Science Monitor 2016;22:2528–50. 27432511 10.12659/MSM.898866 PMC4962757

[18] Ito K Akamatsu N Ichida A Splenectomy is not indicated in living donor liver transplantation. Liver Transpl 2016;22:1526–35. 27253521 10.1002/lt.24489

[19] Lusebrink R Blumhardt G Lohmann R Does concommitant splenectomy raise the mortality of liver transplant recipients? Transpl Int 1994;7 (suppl 1):S634–6. 11271326 10.1111/j.1432-2277.1994.tb01461.x

[20] Samimi F Irish WD Eghteasd B Role of splenectomy in human liver transplantation under modern-day immunosuppression. Dig Dis Sci 1998;43:1931–7. 9753254 10.1023/a:1018822206580 PMC2977917

[21] Shimada M Ijichi H Yonemura Y The impact of splenectomy or splenic artery ligation on the outcome of a living donor adult liver transplantation using a left lobe graft. Hepatogastroenterology 2004;51:625–9. 15143878

[22] Umeda Y Yagi T Sadamori H Preoperative proximal splenic artery embolization: a safe and efficacious portal decompression technique that improves the outcome of live donor liver transplantation. Transpl Int 2007;20:947–55. 17617180 10.1111/j.1432-2277.2007.00513.x

[23] Kelly DM Miller C Understanding the splenic contribution to portal flow: the role of splenic artery ligation as inflow modification in living donor liver transplantation. Liver Transpl 2006;12:1186–8. 16868947 10.1002/lt.20880

[24] Golse N Mohkam K Rode A Surgical management of large spontaneous portosystemic splenorenal shunts during liver transplantation: splenectomy or left renal vein ligation? Transplant Proc 2015;47:1866–76. 26293065 10.1016/j.transproceed.2015.06.019

[25] Lee SD Kim SH Kong SY ABO-incompatible living donor liver transplantation without graft local infusion and splenectomy. HPB (Oxford) 2014;16:807–13. 24467804 10.1111/hpb.12215 PMC4159453

[26] Tronina O Durlik M Wawrzynowicz-Syczewska M Real-world safety and efficacy of ombitasvir/paritaprevir/ritonavir/+dasabuvir ± ribavirin (OBV/PTV/r/+DSV ± RBV) therapy in recurrent hepatitis C virus (HCV) genotype 1 infection post-liver transplant: AMBER-CEE study. Ann Transplant 2017;22:199–207. 28386057 10.12659/AOT.903535 PMC12577526

[27] Heuer M Taeger G Kaiser GM No further incidence of sepsis after splenectomy for severe trauma: a multi-institutional experience of the trauma registry of the DGU with 1,630 patients. Eur J Med Res 2010;15:258–65. 20696635 10.1186/2047-783X-15-6-258 PMC3351995

[28] Tarantino G Scalera A Finelli C Liver-spleen axis: intersection between immunity, infections and metabolism. World J Gastroenterol 2013;19:3534–42. 23801854 10.3748/wjg.v19.i23.3534 PMC3691032

[29] Sun LM Chen HJ Jeng LB Splenectomy and increased subsequent cancer risk: a nationwide population-based cohort study. Am J Surg 2015;210:243–51. 25986002 10.1016/j.amjsurg.2015.01.017

[30] Wu SC Fu CY Muo CH Splenectomy in trauma patients is associated with an increased risk of postoperative type II diabetes: a nationwide population-based study. Am J Surg 2014;208:811–6. 24928333 10.1016/j.amjsurg.2014.03.003

[31] Watters JM Sambasivan CN Zink K Splenectomy leads to a persistent hypercoagulable state after trauma. Am J Surg 2010;199:646–51. 20466110 10.1016/j.amjsurg.2010.01.015

[32] Wells GA Shea B O’Connell D The Newcastle–Ottawa Scale (NOS) for assessing the quality of non-randomized studies in meta-analysis. Available from: www.ohri.ca/programs/clinical_epidemiology/oxford.asp.

[33] Parmar MK Torri V Stewart L Extracting summary statistics to perform meta-analyses of the published literature for survival endpoints. Stat Med 1998;17:2815–34. 9921604 10.1002/(sici)1097-0258(19981230)17:24<2815::aid-sim110>3.0.co;2-8

[34] Tierney JF Stewart LA Ghersi D Practical methods for incorporating summary time-to-event data into meta-analysis. Trials 2007;8:16. 17555582 10.1186/1745-6215-8-16 PMC1920534

[35] Hozo SP Djulbegovic B Hozo I Estimating the mean and variance from the median, range, and the size of a sample. BMC Med Res Methodol 2005;5:13. 15840177 10.1186/1471-2288-5-13 PMC1097734

[36] Badawy A hamaguchi Y Satoru S Evaluation of safety of concomitant splenectomy in living donor liver transplantation. Transpl Int 2017;30:914–23. 28512755 10.1111/tri.12985

[37] Wang H Ikegami T Harada N Optimal changes in portal hemodynamics induced by splenectomy during living donor liver transplantation. Surg Today 2015;45:979–85. 25080864 10.1007/s00595-014-0999-9

[38] Liu KF Zheng H Shen ZY Impact of splenectomy on the hepatic hemodynamics in patients received liver transplantation. Zhonghua Wai Ke Za Zhi 2011;49:154–7. 21426832 10.3760/cma.j.issn.0529-5815.2011.02.012

[39] Wang WL Gao L Liang TB Effects of splenectomy on patients undergoing liver transplantation. Zhonghua Yi Xue Za Zhi 2006;86:1240–3. 16796881

[40] Ye Qi-fa Effect of splenectomy in prognosis of human liver transplantation. Chinese Journal of Bases and Clinics in General Surgery 2008;15:159–61.

[41] Raut V Mori A Kaido T Splenectomy does not offer immunological benefits in ABO-incompatible liver transplantation with a preoperative rituximab. Transplantation 2012;93:99–105. 22094955 10.1097/TP.0b013e318239e8e4

[42] Kishi Y Sugawara Y Akamatsu N Splenectomy and preemptive interferon therapy for hepatitis C patients after living-donor liver transplantation. Clin Transplant 2005;19:769–72. 16313323 10.1111/j.1399-0012.2005.00419.x

[43] Lv Y Lau WY Li Y Hypersplenism: history and current status. Exp Ther Med 2016;12:2377–82. 27703501 10.3892/etm.2016.3683 PMC5038876

[44] Cheng LY Yu J Zhang W Risk factors of persistent thrombocytopenia after adult liver transplantation and prophylactic measures. Zhejiang Da Xue Xue Bao Yi Xue Ban 2014;43:670–7. 25644566 10.3785/j.issn.1008-9292.2014.11.006

[45] Bessler H Mandel EM Djaldetti M Role of the spleen and lymphocytes in regulation of the circulating platelet number in mice. J Lab Clin Med 1978;91:760–8. 641398

[46] Ichikawa N Itano K Shimodaira S Changes in serum thrombopoietin levels after splenectomy. Acta Haematol 1998;100:137–41. 9858790 10.1159/000040888

[47] Dahm F Georgiev P Clavien PA Small-for-size syndrome after partial liver transplantation: definition, mechanisms of disease and clinical implications. Am J Transplant 2005;5:2605–10. 16212618 10.1111/j.1600-6143.2005.01081.x

[48] Ogura Y Hori T El Moghazy WM Portal pressure <15 mm Hg is a key for successful adult living donor liver transplantation utilizing smaller grafts than before. Liver Transpl 2010;16:718–28. 20517905 10.1002/lt.22059

[49] Gruttadauria S Mandala L Miraglia R Successful treatment of small-for-size syndrome in adult-to-adult living-related liver transplantation: single center series. Clin Transplant 2007;21:761–6. 17988271 10.1111/j.1399-0012.2007.00735.x

[50] Goldaracena N Echeverri J Selzner M Small-for-size syndrome in live donor liver transplantation-Pathways of injury and therapeutic strategies. Clin Transplant 2017;31: 10.1111/ctr.12885 27935645

[51] Lee EC Kim SH Park SJ Outcomes after liver transplantation in accordance with ABO compatibility: a systematic review and meta-analysis. World J Gastroenterol 2017;23:6516–33. 29085201 10.3748/wjg.v23.i35.6516 PMC5643277

[52] Rego J Prevost F Rumeau JL Hyperacute rejection after ABO-incompatible orthotopic liver transplantation. Transplant Proc 1987;19:4589–90. 3321625

[53] Hogen R DiNorcia J Dhanireddy K Antibody-mediated rejection: what is the clinical relevance? Curr Opin Organ Transplant 2017;22:97–104. 28060025 10.1097/MOT.0000000000000391

[54] Monteiro I McLoughlin LM Fisher A Rituximab with plasmapheresis and splenectomy in abo-incompatible liver transplantation. Transplantation 2003;76:1648–9. 14702545 10.1097/01.TP.0000082723.02477.87

[55] Matsuno N Nakamura Y Mejit A Long-term follow-up ABO-incompatible adult living donor liver transplantation in cirrhotic patients. Clin Transplant 2007;21:638–42. 17845639 10.1111/j.1399-0012.2007.00702.x

[56] Neumann UP Berg T Bahra M Long-term outcome of liver transplants for chronic hepatitis C: a 10-year follow-up. Transplantation 2004;77:226–31. 14742986 10.1097/01.TP.0000101738.27552.9D

[57] Russo MW Galanko J Beavers K Patient and graft survival in hepatitis C recipients after adult living donor liver transplantation in the United States. Liver Transpl 2004;10:340–6. 15004758 10.1002/lt.20090

[58] Roayaie S Schiano TD Thung SN Results of retransplantation for recurrent hepatitis C. Hepatology 2003;38:1428–36. 14647054 10.1016/j.hep.2003.09.010

[59] Watt KD Lyden ER McCashland TM Poor survival after liver retransplantation: is hepatitis C to blame? Liver Transpl 2003;9:1019–24. 14526394 10.1053/jlts.2003.50206

[60] Morimoto H Ishiyama K Ishifuro M Clinical efficacy of simultaneous splenectomy in liver transplant recipients with hepatitis C virus. Transplant Proc 2014;46:770–3. 24767345 10.1016/j.transproceed.2013.12.034

[61] Maggi U Dondossola D Consonni D Visceral artery aneurysms in liver transplant candidates and in patients after liver transplantation. PLoS One 2011;6:e29544. 22216310 10.1371/journal.pone.0029544 PMC3244466

[62] Cescon M Sugawara Y Kaneko J Restoration of portal vein flow by splenorenal shunt ligation and splenectomy after living-related liver transplantation. Hepatogastroenterology 2001;48:1453–4. 11677985

[63] Samimi F Irish WD Eghtesad B Role of splenectomy in human liver transplantation under modern-day immunosuppression. Dig Dis Sci 1998;33:1931–7. 10.1023/a:1018822206580 PMC2977917 9753254

[64] Xu YT Liu DJ Meng FY Possible benefit of splenectomy in liver transplantation for autoimmune hepatitis. Hepatobiliary Pancreat Dis Int 2014;13:328–31. 24919618 10.1016/s1499-3872(14)60256-3

[65] Umeda Y Yagi T Sadamori H Effects of prophylactic splenic artery modulation on portal overperfusion and liver regeneration in small-for-size graft. Transplantation 2008;86:673–80. 18791439 10.1097/TP.0b013e318181e02d

[66] Humar A Beissel J Crotteau S Delayed splenic artery occlusion for treatment of established small-for-size syndrome after partial liver transplantation. Liver Transpl 2009;15:163–8. 19177447 10.1002/lt.21636

[67] Fung J Wong T Chok K Long-term outcomes of entecavir monotherapy for chronic hepatitis B after liver transplantation: results up to 8 years. Hepatology 2017;66:1036–44. 28370215 10.1002/hep.29191

[68] Ponziani FR Mangiola F Binda C Future of liver disease in the era of direct acting antivirals for the treatment of hepatitis C. World J Hepatol 2017;9:352–67. 28321272 10.4254/wjh.v9.i7.352 PMC5340991

